# Analysis of the *Campylobacter jejuni* Genome by SMRT DNA Sequencing Identifies Restriction-Modification Motifs

**DOI:** 10.1371/journal.pone.0118533

**Published:** 2015-02-19

**Authors:** Jason L. O’Loughlin, Tyson P. Eucker, Juan D. Chavez, Derrick R. Samuelson, Jason Neal-McKinney, Christopher R. Gourley, James E. Bruce, Michael E. Konkel

**Affiliations:** 1 School of Molecular Biosciences, College of Veterinary Medicine, Washington State University, Pullman, Washington, United States of America; 2 Department of Genome Sciences, University of Washington, Seattle, Washington, United States of America; Iowa State University, UNITED STATES

## Abstract

*Campylobacter jejuni* is a leading bacterial cause of human gastroenteritis. The goal of this study was to analyze the *C. jejuni* F38011 strain, recovered from an individual with severe enteritis, at a genomic and proteomic level to gain insight into microbial processes. The *C. jejuni* F38011 genome is comprised of 1,691,939 bp, with a mol.% (G+C) content of 30.5%. PacBio sequencing coupled with REBASE analysis was used to predict *C. jejuni* F38011 genomic sites and enzymes that may be involved in DNA restriction-modification. A total of five putative methylation motifs were identified as well as the *C. jejuni* enzymes that could be responsible for the modifications. Peptides corresponding to the deduced amino acid sequence of the *C. jejuni* enzymes were identified using proteomics. This work sets the stage for studies to dissect the precise functions of the *C. jejuni* putative restriction-modification enzymes. Taken together, the data generated in this study contributes to our knowledge of the genomic content, methylation profile, and encoding capacity of *C. jejuni*.

## Introduction

*Campylobacter jejuni* is an important pathogen causing significant morbidity and mortality. *C*. *jejuni* is a Gram-negative, comma-shaped, microaerophilic bacterium, and is motile by means of unipolar or bipolar flagella. The genus *Campylobacter* was proposed in 1963, separating these bacteria from *Vibrio*-like organisms based on morphology, DNA composition, microaerobic growth requirement, and non-fermentive metabolism [1]. *C*. *jejuni* was first isolated from human feces in 1972 [2]. Human infection, also called campylobacteriosis, often occurs after handling or ingesting food contaminated by raw poultry products. Clinical infection with *C*. *jejuni* presents as diarrhea with blood and leukocytes, fever, nausea, and severe abdominal cramps that occur 2–5 days following ingestion [3, 4]. In recent years, infection rates with *Campylobacter* spp. has been comparable to or has exceeded that of other enteric pathogens, including *Salmonella* spp. and *Shigella* spp. The highest prevalence of *Campylobacter*-mediated disease is among children less than 5 years of age [5]. Trials with human volunteers have demonstrated that the infectious dose of *C*. *jejuni* is as few as 500 organisms, although there is no clear correlation between dose and disease severity. Although campylobacteriosis is usually self-limiting, erythromycin and azithromycin can be used to treat individuals with severe enteritis. Infection with some *C*. *jejuni* isolates correlates with a higher incidence of Guillain-Barré syndrome (GBS), an autoimmune disease and the most common cause of flaccid paralysis in the post-polio era [6]. The association between *C*. *jejuni* infections and GBS is due to molecular mimicry resulting in a cross-reactive immune response between *Campylobacter* lipo-oligosaccharides (LOS) and neural gangliosides [7, 8]. In the United States, treatment of *C*. *jejuni* infections and GBS is estimated to cost $1.2 billion per year.

Advances in sequencing technology have resulted in an increase in the number of *C*. *jejuni* genomes available to researchers [7, 9, 10]. Deciphering the *C*. *jejuni* pan genome, which is comprised of both the core and variable genes, coupled with the epidemiology and ecology data has led to a deeper understanding of the genomic content and how this can influence the environmental niche and transmission of individual isolates. For example, comparative genomic analysis has demonstrated variability in the genomic content of *C*. *jejuni* strains, possibly influenced by the host from which the isolate was recovered (*e*.*g*., human, chicken, ruminant, etc.) and geographical location. More specifically, the genomes of *C*. *jejuni* human isolates are more similar to non-livestock isolates (environmental) than to livestock isolates, as assessed by DNA microarray analysis of whole-genome comparisons of 111 isolates [11]. This type of analysis will also allow for improved risk-based approaches for implementing efficient *C*. *jejuni*-mediated disease control measures in the environment, at the farm, and in retail consumer meat [12, 13].

Methylation of the genome plays important biological roles in bacteria. Several decades ago it was determined that methylation is an important component of restriction-modification (RM) systems that prevent foreign DNA, including phage DNA, from integrating into the bacterial genome. The DNA in the host is marked through sequence-specific methylation of adenine and cytosine nucleotides. Most RM systems fall into one of four categories, Types I—IV, although non-classical RM systems have been discovered that are not as easily categorized [14]. Classification is based on the number of genes/subunits, methylation and restriction activities performed, co-factors required for activity, recognition sequence, cleavage site, and mechanism of cleavage. Type I RM systems are composed of three different enzymes, designated R, S or M, which are responsible for endonuclease activity, determining the sequence specificity of methylation and cleavage, and methylation of the DNA substrate, respectively. The recognition sequence is both bipartite and asymmetrical with cleavage occurring at variable locations within approximately 1 kilobase from the recognition site. Methylation usually occurs at the N^6^ position of adenines within the recognition site. Type I RM systems are widespread in prokaryotes, including *C*. *jejuni*, where 53 out of 73 *C*. *jejuni* strains tested contained Type I RM loci [15]. Type II RM systems are the most characterized RM systems and are usually comprised of separate restriction endonuclease and methylase enzymes, although there are examples of both activities being performed by a single enzyme. The restriction endonuclease component usually forms a homodimer, while the methylase is a monomer. Both enzymes recognize an identical DNA sequence, which is typically a 4–8 base pair palindrome. Type III RM systems are comprised of two separate enzymes and recognize 5–6 base pair asymmetric sequences, with cleavage occurring at a fixed location within approximately 25 base pairs of the recognition sequence. Type IV RM systems do not modify DNA and only cleave DNA that contains methylated, hydroxymethylated, or glucosyl-hydroxymethylated cytosines. Other methylases include Dam and Dcm. Dam methylates the N^6^ position of adenine in the sequence GATC, while Dcm methylates cytosines at the C^5^ position of the sequences CCAGG and CCTGG. In addition to these functions, methylation in bacteria has also been shown to influence DNA replication, DNA repair, gene transcription, the transfer of plasmids, and mutations in methylases influence virulence [16].

The *C*. *jejuni* F38011 strain was isolated from an individual with bloody diarrhea. *C*. *jejuni* F38011 strain has been shown to efficiently colonize chickens and cause disease in piglets and mice [17–19]. To better understand the genomic content and proteomic capacity of this strain compared to other *C*. *jejuni* strains, we have sequenced the genome and determined the proteome of the *C*. *jejuni* F38011 strain using Pacific Biosciences (PacBio) Single-Molecule Real-Time (SMRT) sequencing technology and LC-MS/MS, respectively. The results of this study demonstrate that the use of new technologies can illuminate differences between genomes and possibly uncover unique features.

## Materials and Methods

### Bacterial strains and growth conditions

The *C*. *jejuni* F38011 clinical strain was used throughout this study. The strain was cultured under microaerobic conditions (85% N_2_, 10% CO_2_, 5% O_2_) on Mueller-Hinton (MH) agar plates supplemented with 5% citrated bovine blood (MHB) at 37°C and passaged to a fresh plate at least once every 48 h. These conditions are optimal for *C*. *jejuni* growth. Assays were performed with *C*. *jejuni* grown on MH agar plates for 48 h and diluted in PBS.

The *C*. *jejuni* F38011 CJH00185 deletion mutant (annotated Cj0031 in NCTC 11168) was generated by double-crossover recombination. The *C*. *jejuni* NCTC 11168 Cj0031 ORF was previously annotated as two genes; however, it has been re-annotated as a single ORF at the time of this study. The PCR primers used for amplifying CJH00185 are 5’-GGGAACAAAAGCTGGAGCTCGTTTGAGTGATATAAATATATTTGATGC-3’ and 5’-AAGGAACACCCGCGGTCTTTTTCATTTAGCAAAGTGAAATGC-3’ to amplify the upstream fragment and 5’-CGGGCCCCCCCTCGAGGACATAAAGCAAATCCAACTAAACC-3’ and 5’-CGGGCCCCCCCTCGAGGACATAAAGCAAATCCAACTAAACC-3’ to amplify the downstream fragment. The chloramphenicol resistance cassette was PCR amplified using primers 5’-AAGGAACACCCGCGG-3’ and 5’-GCGCAGATCCCGCGG-3’ from a previously constructed pBSK vector template. The CJH00185 upstream, CJH00185 downstream, and the amplified chloramphenicol resistance cassette fragments were ligated into the pBSK-kan2 vector, which was linearized with SacI and XhoI, in a 4-way infusion reaction (Clontech Laboratories, Inc., Mountain View, CA). The upstream and downstream fragment primers contain the overlapping sequences for the cloning reaction. The resulting pBSK-kan vector, which harbors the chloramphenicol resistance cassette flanked by 5’ and 3’ fragments for the deletion of CJH00185, was transformed into the *C*. *jejuni* wild-type strain and chloramphenicol resistant colonies were isolated. Deletion of the CJH00185 gene in the *C*. *jejuni* CJH00185 mutant was confirmed by PCR and genomic sequence analysis.

### Genome Sequencing, Methylation and Bioinformatics

DNA was isolated from the *C*. *jejuni* F38011 wild-type strain and the CJH00185 mutant following standard laboratory procedures for phenol/chloroform extraction. The DNA libraries were prepared following PacBio guidelines and sequenced on SMRT cells using Pacific Biosciences RS sequencing technology (Pacific Biosciences, Menlo Park, CA) at Washington State University. Two separate libraries were constructed for sequencing: a small fragment library used to target high quality circular consensus reads generated during polymerase strand displacement mode and a large fragment library targeting continuous long reads. Small fragments were obtained by shearing 5 μg of genomic DNA through the small orifice ruby of a Hydroshear Plus (Digilabs) for 20 cycles at speed code seven. Small fragment library preparation was performed using the Pacific Biosciences DNA Template Prep Kit 2.0 (250 bp to < 3 kb range). Size selection and library purification was performed with 0.6X AMPure beads (Beckmann-Coulter Genomics). The resulting library had a peak size of 2.7 kb and ranged from 900 bp to 9 kb. This library was bound to C2 DNA polymerase, loaded into four SMRT cells, and polymerization was observed using two 45 min movies for each cell. Large fragment library preparation was performed by shearing 5 μg of genomic DNA using a Hydroshear large orifice ruby for 20 cycles at speed code 20. The large fragment library preparation was performed using the Pacific Biosciences DNA Template Prep Kit 2.0 (3 to 10 kb range). Size selection and library purification was performed using 0.45X AMPure beads (Beckmann-Coulter Genomics). The resulting library had a peak size of 16.5 kb and ranged from 4 to 35 kb. The large fragment library was bound to C2 DNA polymerase, mag-bead loaded into five SMRT cells, stage started, and observed using a single 2 h movie for each cell. The 13 movies generated 232k reads with an average length of 3743 bp.

Assembly was performed using the RS_ Hierarchical Genome Assembly Process (HGAP)_Assembly.1 protocol incorporated into SMRT Analysis v2.0. A preassembled correction contained 48 Mb of long and 628 Mb of short reads using a software calculated minimum seed read length of 9624 bp generating 4272 reads with an N50 length of 10,548 bp. The preassembled reads were assembled using Celera2.0 and polished with Quiver into two contigs with perfect circularizing overlap (1,691,939 bp and 19,277 bp). Mapping concordance of the final assembly was 99.9999. The RS_Modification_and_Motif_Analysis.1 protocol of the SMRT analysis v2.0 was used to identify methylation and the corresponding motifs of the responsible DNA methylases.

Genome annotation was performed by the National Center for Biotechnology Information (NCBI) Prokaryotic Genomes Automatic Annotation Pipeline and is published as *Campylobacter jejuni* F38011 with ‘CJH’ locus tags (BioSubmission: SUB358967; BioProject ID: PRJNA222831). The *C*. *jejuni* F38011 genome was visualized using CGView [20]. *C*. *jejuni* F38011 and NCTC 11168 genomic DNA sequences (accession number 700819) were compared using Artemis Comparison Tool (ACT), as described elsewhere [21]. Consensus sequence logos were created as described elsewhere [22].

### Proteomic Analysis

A *C*. *jejuni* whole cell lysate was digested with trypsin. The resulting peptides were analyzed by LC-MS/MS, using a Waters nanoAcquity UPLC coupled to a Thermo Velos-FTICR mass spectrometer [23]. Peptides were loaded onto a 3 cm x 100 μm inner diameter fused silica trap column packed with a stationary phase consisting of MichromMagic C18, 5 μm diameter, 200 A pore size particles (Bruker) with a flow rate of 2 μL/min of mobile phase consisting of 98% solvent A (H_2_O containing 0.1% formic acid) and 2% solvent B (ACN containing 0.1% formic acid) for 10 minutes. Peptides were then fractionated over a 60 cm x 75 μm inner diameter fused silica analytical column packed with Michrom Magic C18, 5 μm diameter, 100 A pore size particles by applying a linear gradient from 95% solvent A, 5% solvent B to 65% solvent A, 35% solvent B over 120 minutes at a flow rate of 300 nL/min. Eluting peptide ions were ionized by electrospray ionization by applying a positive 2 kV potential to a laser pulled spray tip at the end of the analytical column. The Velos-FTICR mass spectrometer was operated using data dependent method consisting of a high resolution MS^1^ measurement in the ICR cell at 50K resolving power followed by low resolution MS^2^ scans in the ion trap on the 20 most intense ions with charge state of 2+ or greater. MS^2^ settings included a normalized collision energy of 35, and isolation width of 2 *m/z*, and an activation time of 10 ms. Ions selected for MS2 were placed on a dynamic exclusion list for 45 seconds. A total of four biological samples were analyzed in triplicate.

Tandem mass spectra data were searched against a protein database for *C*. *jejuni* strain 11168 (UniProt entry CAMJE) containing forward and reverse sequences (3246 total protein sequences) as well as a database containing forward and reverse sequences for *C*. *jejuni* strain F38011 (3368 total protein sequences), using SEQUEST (version UWPR2012.01). SEQUEST search parameters included: consideration of only fully tryptic peptide sequences, a 25 ppm precursor ion mass tolerance, a 0.36 Da fragment ion mass tolerance, the static modification of carbamidomethylation of Cys residues (57.021464 Da), and the variable modification of oxidation on Met residues (15.9949 Da). A 1% false discovery rate (FDR) threshold was applied to the resulting peptide spectrum matches. All proteins were analyzed and sorted based on subcellular locations using PSORTb v.2.0 [24]. Enzymatic functional class annotations were obtained from the UniProt database for 485 enzymes identified in this study [25].

### Tissue culture

INT 407 human intestinal epithelial cells (ATCC CCL6; American Type Culture Collection, Manassas, VA, USA) were cultured as previously described [26].

### Binding and internalization assays

Determination of *C*. *jejuni* adherence and internalization by INT 407 cells was assessed as previously described [27]. Assays were performed using a multiplicity of infection of 50–500 and reproduced at least 3 times. Statistical analyses were performed using GraphPad Prism 6 (La Jolla, CA. USA) and statistical significance (*P* ≤ 0.05) was determined by one-way analysis of variance (ANOVA) using Tukey’s post-test.

### Growth characterization of *C*. *jejuni* strains and survival in deoxycholate (DOC)

Overnight MHB plate cultures of *C*. *jejuni* strains were used to inoculate fresh MH broth, with and without 0.05% DOC concentration, at an OD_540_ of 0.01 and bacterial density was monitored at the indicated time points using a spectrophotometer. Survival was assessed by plating 10-fold serial dilutions of the broth cultures on MHB plates, at the indicated time points, and colonies were counted to determine CFU/ml. Statistical analyses were performed using GraphPad Prism 6 and statistical significance (*P* ≤ 0.05) was determined by ANOVA using Tukey’s post-test.

## Results and Discussion

**Fig 1:**
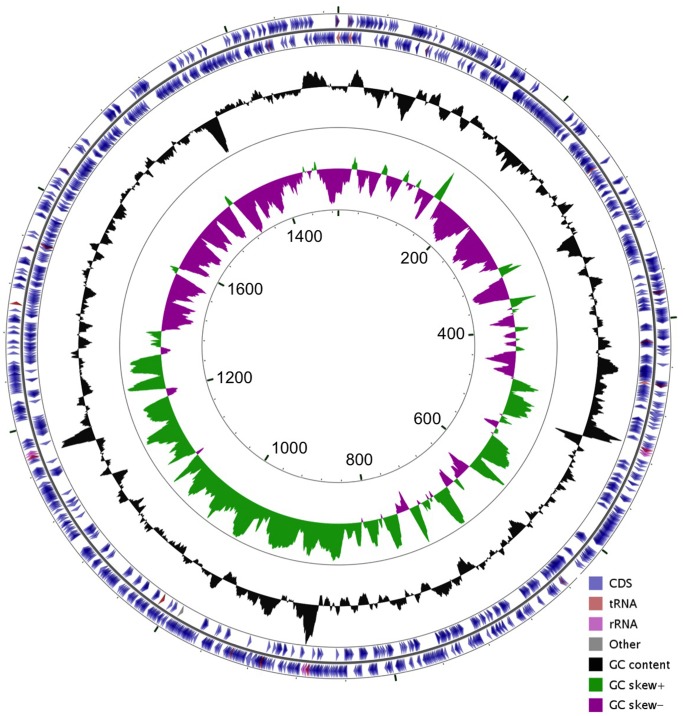
Genomic map of the *C*. *jejuni* F38011 strain. A 1.69 Mbp circular chromosome of the *C*. *jejuni* F38011 strain, beginning with DnaA and encoding a putative 1613 CDSs. Direction of the predicted protein coding sequences (CDS), transfer RNAs (tRNA), ribosomal RNAs (rRNA), mol.% (G+C) content, and GC skew are indicated.

**Fig 2:**
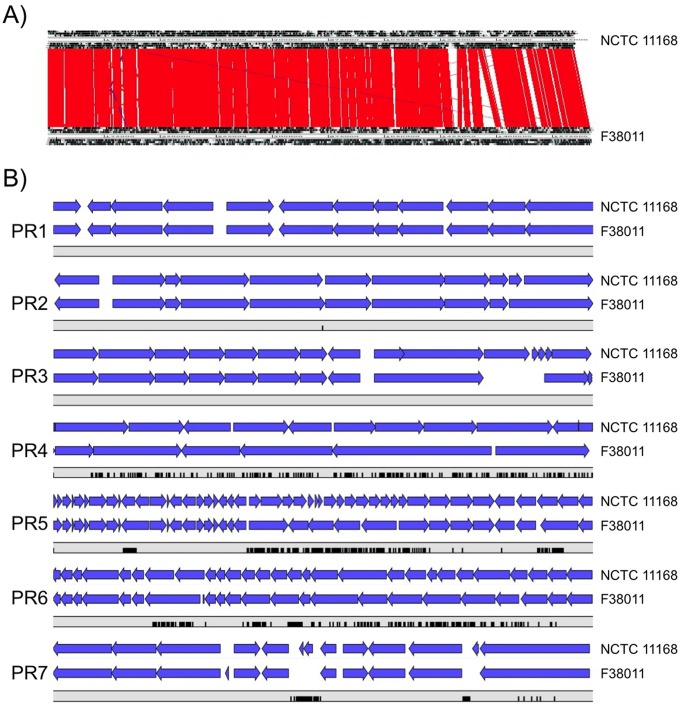
The *C*. *jejuni* F38011 genome shares similarity to the NCTC 11168 genome. Panel A) Analysis of the *C*. *jejuni* F38011 and NCTC 11168 genomes using Artemis Comparison Tool (ACT) shows the gene order and organization of the *C*. *jejuni* F38011 genome is similar to the NCTC 11168 strain. Blocks of similarity are indicated with red and inversions are indicated with blue between strains. Panel B) Comparison of the seven hypervariable plasticity regions (PR1—PR7) in the genomes of the *C*. *jejuni* F38011 and NCTC 11168 strains. Genes are shown in blue with the gray bar below each PR indicating the gap fraction of pairwise alignment with black columns representing absence of genetic information in the *C*. *jejuni* F38011 strain. PR parameters were determined as described elsewhere [33].

The *C*. *jejuni* F38011 clinical strain (biotype I, serotype 90) was isolated from an individual with bloody diarrhea [28, 29]. Given that this *C*. *jejuni* strain has been used for more than two decades to dissect the virulence proteins and associated factors necessary for *Campylobacter* pathogenesis, we sought to more fully characterize this strain. The genome of the *C*. *jejuni* F38011 strain was sequenced using Pacific Biosciences sequencing technology. The *C*. *jejuni* F38011 genome was determined to be 1.69 Mb (1,691,939) with 1613 predicted protein coding sequences (CDSs) (Fig. 1). The genome was sequenced with 100% of bases called, forming a single circular genome from library preparation (99.9999% consensus accuracy) and average nucleotide coverage of 396. No plasmids were identified. The mol.% (G+C) content was 30.5% and the change in GC skew demonstrates origin of replication for the *C*. *jejuni* F38011 strain, as observed elsewhere [30]. Genomic regions associated with the largest changes in mol.% (G+C) content reflect points in the genome encoding tRNAs or rRNAs. Using the Artemis Comparison Tool (ACT), the genomes of the *C*. *jejuni* F38011 and NCTC 11168 strains were compared (Fig. 2). *C*. *jejuni* NCTC 11168 is 1.6 Mb (1,641,481) with a mol.% (G+C content) of 30.5% [31]. There is substantial synteny between strains and similarity in mol.% (G+C) content, even though the *C*. *jejuni* F38011 genome is greater in size. Furthermore, the *C*. *jejuni* F38011 strain contained 122 putative CDSs with little similarity to genes found in NCTC 11168 strain; however, the *C*. *jejuni* F38011 strain also lacked 58 CDSs present in the NCTC 11168 genome (S1 Table). Many of the CDSs unique to either strain fall within the hypervariable plasticity regions of the organism, as discussed below. Additionally, the *C*. *jejuni* F38011 strain does not harbor plasmids that confer resistance to antibiotics, including the tetracycline resistance plasmid found in *C*. *jejuni* 81–176 (pTet plasmid) [32].

The pan genome, which is comprised of both the core and variable genes, was evaluated in the *C*. *jejuni* F38011 strain. Previous comparative genomic hybridization analysis using microarrays to assess the genomic content of 11 *C*. *jejuni* clinical isolates identified the common or core genes despite extensive genetic variability between isolates [34]. The common genes, which were nearly 84% of the total genes, encoded proteins involved in metabolic, biosynthetic, cellular, and regulatory processes. The regions of the genome that were variable included genes encoding the biosynthesis and modification of the flagella, LOS and capsular polysaccharide. It was later determined that the variable regions were generally found in seven clusters termed hypervariable plasticity regions (PR; PR1—PR7) [33]. The genetic content of PR1 encodes proteins associated with molybdenum reduction and transport and biotin synthesis; PR2 contains putative membrane transporters and hypothetical proteins; PR3 contains ABC transporters, membrane, and hypothetical proteins; PR4 encodes N-Acetyl Neuraminic Acid (NANA) synthase genes; PR5 contains components of the LOS biosynthesis and flagellar genes (including posttranslational modification); PR6 harbors components responsible for capsule biosynthesis; and PR7 contains genes that encode membrane proteins. Using the PR parameters determined by Pearson and colleagues [33], we compared each variable region of the *C*. *jejuni* F38011 strain to the *C*. *jejuni* NCTC 11168 strain (Fig. 2). While PR1, PR2, PR3 were nearly identical between these two *C*. *jejuni* strains, differences were observed in PR4, PR5, PR6, and PR7. Others have also observed considerable genomic diversity between *C*. *jejuni* isolates recovered from a similar biological source [35, 36]. Additional research is needed to assess the contribution of the gene products located within these hypervariable regions to the phenotypic variation, microbial adaptation to an environmental niche, and/or an isolate’s virulence.

### Methylation motifs in the *C*. *jejuni* F38011 genome

**Fig 3:**
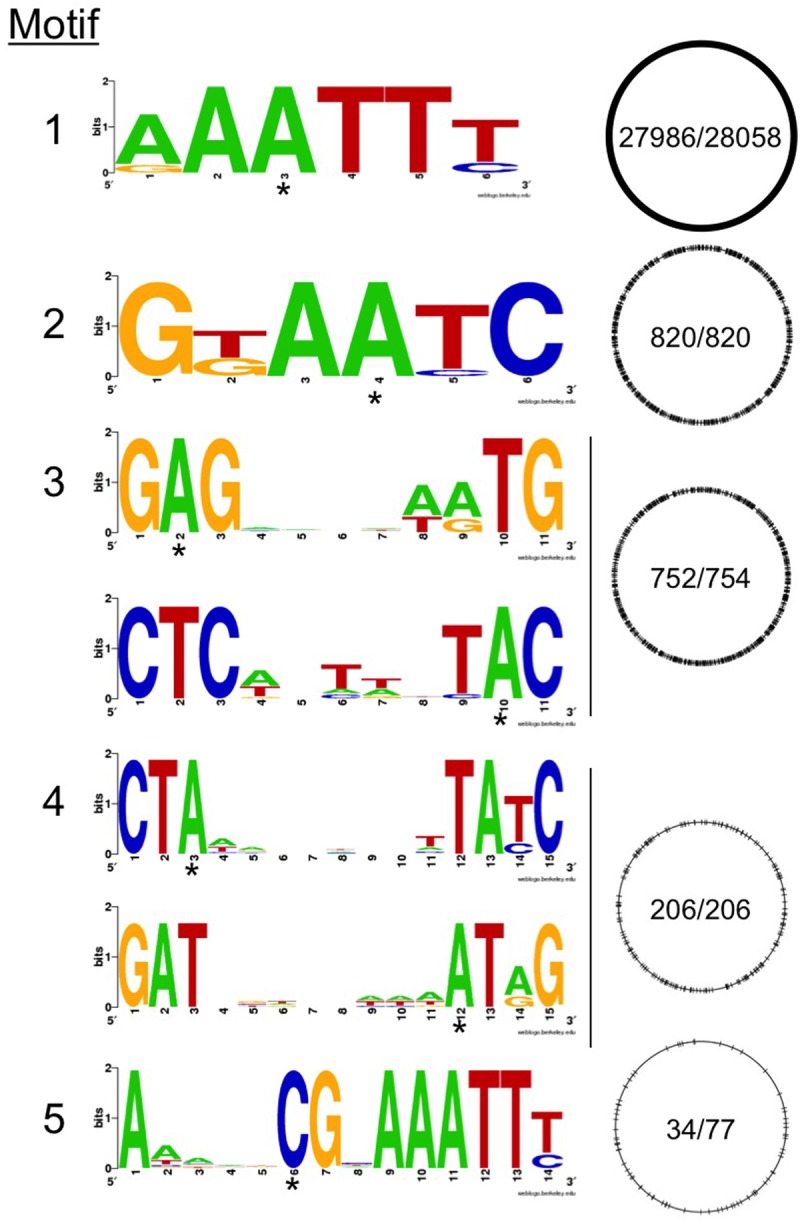
The methylome of *C*. *jejuni* F38011 contains 5 dominant methylation motifs. The methylation consensus sequences identified by PacBio with adenine methylations found in motifs 1, 2, 3, and 4 (motifs 1, 2 and 4 have a partner motif; RAATTY partner motif not shown) and cytosine methylation found in motif 5. Consensus sequences for each motif is represented as logos, where the height of each stack indicates conservation of sequence (bits) and the height of the symbols represent the relative frequency of the base. An asterisk below a base indicates the modified nucleotide in each consensus sequence. The consensus sequence on the circular genome is indicated with a black line. The numbers within each genome represent methylated sequences compared to the total number of each identified consensus sequence.

PacBio sequencing is a reliable and robust method to determine modified bases due to changes in the kinetics of the DNA polymerase as it incorporates the appropriate nucleotide opposite a modified base [37]. DNA methylation was determined in the *C*. *jejuni* F38011 strain using the PacBio RS sequencing platform and total base modifications were detected during SMRT sequencing. The modified bases were clustered into five consensus sequences (plus additional modified bases that were not clustered) and were distributed throughout the genome (Fig. 3). The motifs identified were consistent with two types of methylation: four motifs are recognized by ^m6^A methyltransferases (N-6 adenine-specific DNA methylases) and a single motif is recognized by ^m4^C methyltransferases (N-4 cytosine-specific DNA methylases). Motifs 1, 3 and 4 contained bases methylated on both strands, whereas bases on motifs 2 and 5 were modified on only one strand at each site. The dominant ^m6^A methylation motifs were abundant and nearly completely methylated, as the percent of methylated motifs detected range from 99.5 to 100% for motifs 1, 2, 3 and 4. The ^m4^C modification (motif 5) had a consensus sequence of ANNNNCGNAAATTY and was methylated on 34 of 77 motifs (44.2% percent of motifs detected). The mean modification quality value (QV = 122) and the mean coverage was 188, which is necessary for a confident base modification call at this level of coverage (Table 1). However, because the modification signatures can vary due to the dynamic nature of the kinetic signal, we cannot rule out the possibility that Motif 5 is a false positive motif overcalled by PacBio software, as described elsewhere [38].

**Table 1:** Methylation motif analysis of *C*. *jejuni* F38011 wild-type isolate, as assessed by PacBio sequencing.

Motif	Consensus Sequence	Modified Position	Modification Type	% Motifs Detected	# of Motifs Detected	# of Motifs in Genome	Mean Modification QV	Mean Motif Coverage	Partner Motif
1	RAATTY	3	^m6^A	99.74	27986	28058	251.3	167	RAATTY
2	GKAAYC	4	^m6^A	100	820	820	274.6	179.4	
3	GAGNNNNNRTG	2	^m6^A	99.73	752	754	238.9	181.3	CAYNNNNNCTC
4	CTANNNNNNNNTAYC	3	^m6^A	100	206	206	267.7	191.9	GRTANNNNNNNNTAG
5	ANNNNCGNAAATTY	6	^m4^C	40.26	31	77	122	188.1	
	*Not Clustered*	0		0.47	16068	3398079	43.9	191.5	

The *C*. *jejuni* F38011 strain genome was assessed by REBASE (www.rebase.neb.com) to determine putative *C*. *jejuni* F38011 enzymes (Cje) involved with each motif and for comparisons with known modification systems (Table 2). Motif 1 (RAATTY) identified in the *C*. *jejuni* F38011 genome is associated with a Type II restriction-modification system common to Gram-positive and Gram-negative bacteria. REBASE analysis indicated that the RM enzyme pair CjeF38011P and M.CjeF38011I is predicted to be responsible for RAATTY modification. Motif 3 (GAGNNNNNRTG) has been identified in *C*. *jejuni* strain RFL1 and is digested by the restriction enzyme CjuII. The Type II RM system responsible for Motif 3 likely consists of CjeF38011ORFDP, based on 87% identity to CjeNII. Motif 4 (CTANNNNNNNNTAYC) is a Type I RM and predicted to be modified by the RM enzymes CjeF38011IIP, S.CjeF38011II, and M.CjeF38011II. Motifs 2 (GKAAYC) and 5 (ANNNNCGNAAATTY) are both predicted to be part of the Type II restriction modification system. CjeF38011ORFCP is the likely gene candidate responsible for modification at the consensus sequence GKAAYC based on 98% identity to CjeNIII (GKAAYG). There are two candidate enzymes that may be responsible for modification at Motif 5 (ANNNNCGNAAATTY): 1) M.CjeF38011ORFEP and 2) CjeF38011ORFAP. M.CjeF38011ORFEP shares 98.9% amino acid identity with CJE0220 from *C*. *jejuni* RM1221, as judged by BLAST analysis, and is annotated as a DNA adenine methylase (http://microbesonline.org/). The second candidate enzyme, CjeF38011ORFAP, shares similarity with Cj0031 (originally designated Cj0031/Cj0032) from *C*. *jejuni* NCTC 11168 that contains a hypervariable homopolymeric tract. CjeF38011ORFAP may or may not be a member of a new class of split DNA methyltransferases [39] (Dr. Richard J. Roberts, personal communication). The split DNA methyltransferases usually consist of 2 or 3 subunit enzymes necessary for full activity, although there is evidence that they demonstrate partial activity with just one subunit [40]. Furthermore, there are members of this family that are not split and are single polypeptide enzymes including CjeFV, which recognizes GGRCA but only as a methylase [41].

**Table 2:** Putative *C*. *jejuni* F38011 Restriction Modification systems.

Type	Gene	Enzyme Name	Predicted Recognition Sequence	Genome Coordinates	CDS
I	R	CjeF38011IIP	CTANNNNNNNNTAYC	1527342–1529651	Cjh08075
I	S	S.CjeF38011II	CTANNNNNNNNTAYC	1532872–1534065	Cjh08090
I	M	M.CjeF38011II	CTANNNNNNNNTAYC	1535256–1536740	Cjh08100
II	RM	CjeF38011ORFAP	ANNNNCGNAAATTY	46431–49921	Cjh00185
II	R	CjeF38011IP	RAATTY	210217–210489	Cjh01080
II	M	M.CjeF38011I	RAATTY	210489–211592	Cjh01085
II	RM	CjeF38011ORFCP	GKAAYC	651959–655711c	Cjh03470
II	RM	CjeF38011ORFDP	GAGNNNNNRTG	992549–996643c	Cjh05290
II	M	M.CjeF38011ORFEP	ANNNNCGNAAATTY	1361489–1362409c	Cjh07145
IV	R	CjeF38011McrBP		143327–145267	Cjh00710
IV	R	CjeF38011McrCP		145254–146591	Cjh00715

### Presence of the ANNNNCGNAAATTY motif in *C*. *jejuni* strains and analysis of the CJH00185 sequence

REBASE predicted that the gene CJH00185 (CjeF38011ORFAP) is one of two putative Type IIG methylases in *C*. *jejuni* F38011 that could be responsible for DNA methylation of the N-4 cytosine (^m4^C) at the ANNNNCGNAAATTY motif. All of these sites also contain the RAATTY motif. Considering this ^m4^C motif was previously not recognized in *C*. *jejuni* as a site of DNA methylation, we first inspected the genomes of three additional *C*. *jejuni* strains (NCTC 11168, 81–176, and RM1221) for the presence of the ^m4^C methylated motif. The *C*. *jejuni* NCTC 11168 and 81–176 strains were recovered from the stools of individuals with campylobacteriosis whereas the *C*. *jejuni* RM1221 strain was recovered from the skin of a chicken [42–44]. All three *C*. *jejuni* strains shared 23 of the 34 genomic regions associated with the ^m4^C motif in the *C*. *jejuni* F38011 strain (Table 3). The only ^m4^C site that was unique to the *C*. *jejuni* F38011 genome was associated with a hypothetical protein (CJH01365).

**Table 3:** ^m4^C Methylation motif (ANNNNCGNAAATTY) associated with CDSs in 4 *C*. *jejuni* strains.

F38011 CDS	F38011	NCTC11168	81176	RM1221
Cjh01365	X			
Cjh03960	X	x		
Cjh07705	X	x		
Cjh04175	X	x		x
Cjh02885	X	x	x	
Cjh06100	X	x	x	x
Cjh00635	X	x	x	x
Cjh01015	x	x	x	x
Cjh08650	x	x	x	x
Cjh06140	x	x	x	x
Cjh04030	x	x	x	x
Cjh00185*	x	x	x	x
Cjh000480	x	x	x	x
Cjh00975	x	x	x	x
Cjh04615	x	x	x	x
Cjh03835	x	x	x	x
Cjh01715	x	x	x	x
Cjh04960	x	x	x	x
Cjh03470	x	x	x	x
Cjh04685	x	x	x	x
Cjh05175	x	x	x	x
Cjh02615	x	x	x	x
Cjh00950	x	x	x	x
Cjh02975	x	x	x	x
Cjh08880	x	x	x	x
Cjh03400	x	x	x	x
Cjh08420	x	x	x	x
Cjh03600	x	x	x	x
Cjh01665	x		x	x
Cjh01310	x		x	x
Cjh02300	x		x	x
Cjh04945	x		x	x
Cjh09850	x		x	
Cjh02865	x		x	

* Indicates putative enzyme responsible for m4C modifications

Inspection of the CJH00185 sequence revealed a frameshift mutation within the 3491 nucleotides that splits the sequence into two ORFs and could prevent the translation of the full-length CDS of the CjeF38011ORFAP (S1 Fig.). Noteworthy is that the Cj0031 gene of *C*. *jejuni* NCTC 11168, which is a putative homologue of CJH00185, contains a hypervariable homopolymeric tract [41]. In contrast to the Cj0031 gene that has a polymorphic poly-G tract within the CDS (8–10 bases), we identified a tract of four bases in a similar location within the CJH00185 gene.

### Proteomic analysis of the *C*. *jejuni* F38011 strain

**Fig 4:**
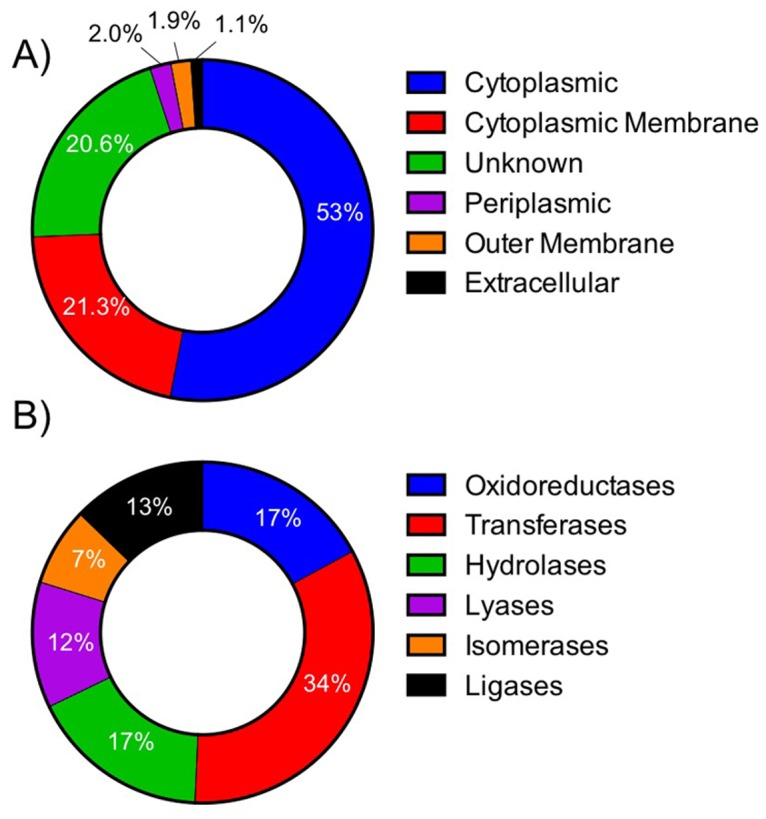
Analysis of the proteins synthesized by *C*. *jejuni* F38011. Panel A) Full proteomic analysis of *C*. *jejuni* F38011 proteins identified by LC-MS/MS analysis that scored greater than the 1% false discovery rate (FDR) arranged by subcellular localization, as assessed by PSORT. Panel B) Analysis of 485 enzymes grouped by functional classes synthesized by *C*. *jejuni* F38011, as derived from UniProt.

Total protein was assessed in the *C*. *jejuni* F38011 strain to determine the putative restriction-modification enzymes that are synthesized. The proteins produced by the *C*. *jejuni* F38011 strain were collected after 48 h growth in rich broth medium and analyzed by LC-MS/MS. A total of 1336 proteins (82% of the 1613 predicted CDSs) were identified when mapped to *C*. *jejuni* NCTC 11168 (listed in S2 and S3 Tables). Interestingly, peptides matched uniquely to more than 30 CDSs in the *C*. *jejuni* F38011 database compared to NCTC 11168 (S3 Table). Only the proteins with peptides identified at less than 1% false discovery rate were included in the analysis. The identified proteins were assessed by the PSORTb v.2.035 program (http://www.psort.org/psortb/index.html) to classify subcellular locations. The subcellular localization of proteins was predicted to group into three major classes: cytoplasmic proteins (53%), cytoplasmic membrane proteins (21%), and proteins of unknown location (21%) (Fig. 4). Comparing the 1336 proteins identified against the UniProt entry for the *C*. *jejuni* 11168 strain, we identified 485 enzymes that were grouped into six functional classes, with transferases (34%) representing the most abundant group (Fig. 4).

Consistent with the results of genomic sequence and methylation profiles, peptide fragments corresponding to the *C*. *jejuni* putative RM enzymes (Table 2) were identified by LC-MS/MS. For example, four peptide fragments corresponding to the deduced amino acid sequence of CjeF38011ORFAP protein were detected (S3 Table). However, the CjeF38011ORFAP peptide fragments identified were localized within the amino-terminal region of the CDS (S1 Fig.). While this finding indicates that CJH00185 is expressed and a protein is synthesized, it is not possible to determine the true CDS from the LC-MS/MS analysis. In summary, the LC-MS/MS data indicates that the RM enzymes listed in Table 2 are synthesized.

### Preliminary characterization of a *C*. *jejuni* F38011 CJH00185 deletion mutant

We chose to assess the contribution of the gene CJH00185, as it is predicted to encode a putative Type II RM methylase. A *C*. *jejuni* CJH00185 mutant was constructed in which the entire CDS was deleted and the mutant was analyzed by PacBio sequencing. The *C*. *jejuni* CJH00185 mutant mapped to the wild-type reference strain at all positions (except where the gene deletion occurred), with 100% of bases called with 99.99% consensus accuracy and an average nucleotide coverage of 342.4. Interestingly, the modified bases found in the mutant clustered into only four consensus sequences (Table 4). Specifically, PacBio SMRT analysis did not identify modified bases corresponding to motif 5 in the CJH00185 mutant. However, based on the data in-hand, we cannot rule out the possibility that the PacBio SMRT analysis failed to identify motif 5 in the *C*. *jejuni* CJH00185 mutant due to the relative difficulty in identifying partially methylated DNA.

**Table 4:** Methylation motif analysis of *C*. *jejuni* F38011 ΔCjh_00185 mutant, as assessed by PacBio sequencing.

Motif	Consensus Sequence	Modified Position	Modification Type	% Motifs Detected	# of Motifs Detected	# of Motifs in Genome	Mean Modification QV	Mean Motif Coverage	Partner Motif
1	RAATTY	3	m6A	99.28	27603	27804	213.5	158.1	RAATTY
2	GKAAYC	4	m6A	99.63	810	813	228.6	160.8	
3	GAGNNNNNRTG	2	m6A	98.78	729	738	205.5	164.2	CAYNNNNNCTC
4	CTANNNNNNNNTAYC	3	m6A	99.51	205	206	222.8	167.2	GRTANNNNNNNNTAG
	*Not Clustered*	0		0.26	8739	3353373	42.7	167.5	

**Fig 5:**
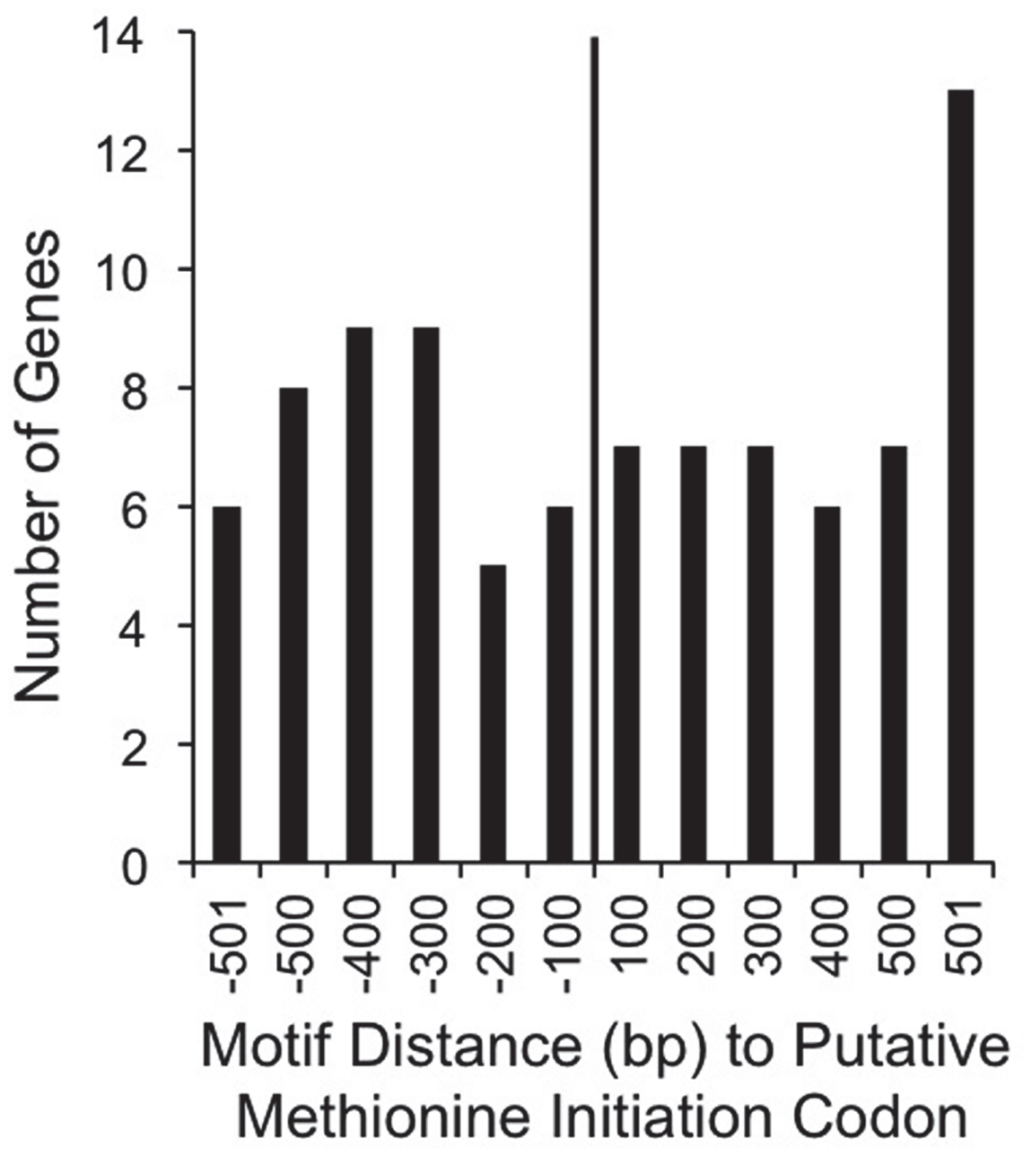
Genomic sites of methyl-cytosine modification is not correlated near putative methionine initiation codons. The methyl-cytosine consensus sequence is distributed throughout the genome with no correlation of proximity near the putative methionine initiation codon.

Although many ^m4^C modifications protect bacteria from foreign DNA, the majority of ^m4^C modifications do not have known biological roles [45]. We initially hypothesized that ^m4^C methylation could play a role in gene regulation. However, the methyl-cytosine consensus sequence was distributed throughout the entire genome without statistical correlation to the nearest putative methionine initiation codon (Fig. 5). Therefore, we have no evidence for methylation altering gene expression in *C*. *jejuni*. Another possibility is that the methylation influences *C*. *jejuni* fitness, as has been shown for *Brucella*, *Escherichia coli*, *Haemophilus*, *Salmonella*, *Vibrio*, and *Yersinia* [16]. To test this possibility, several phenotypic assays were performed. Malik-Kale and colleagues identified a set of *C*. *jejuni* genes upregulated in the presence of a physiologically relevant concentration of the bile acid DOC [46]. More specifically, microarray data from *C*. *jejuni* grown in the presence of DOC revealed that multiple genes were upregulated compared to growth without DOC [46]. *C*. *jejuni* inoculation of growth medium supplemented with DOC increases the kinetics of cellular invasion and increases the secretion and delivery of key virulence proteins to the cytosol of host cells, as compared to growth in non-supplemented growth medium. Thus, we characterized the *C*. *jejuni* CJH00185 deletion mutant in assays with and without physiological concentrations of DOC (0.05%) and determined whether this mutant is capable of binding to and invading epithelial cells. Bacterial growth rates were similar between strains in MH broth with and without 0.05% DOC (S2 Fig.). Bacterial survival over time was also similar between strains in MH broth with and without 0.05% DOC, as determined by CFU analysis (S2 Fig.). Furthermore, adherence to and invasion of INT 407 cells by *C*. *jejuni* following 30 and 180 min incubation period, respectively, demonstrated no statistical difference between the wild-type and the mutant isolate (S2 Fig.). Thus, the *C*. *jejuni* F38011 CJH00185 mutant is phenotypically indistinguishable from the wild-type strain in these assays.

### Summary

The goal of this study was to analyze the genomic content, methylome profile, and proteomic capacity of the *C*. *jejuni* F38011 clinical strain *to gain insight into* microbial processes. In this study, we have further characterized the *C*. *jejuni* F38011 strain using PacBio's SMRT sequencing coupled with methylome analysis. While this sequencing method provides insight into the DNA methylation status of the *C*. *jejuni* genome, additional work is necessary to confirm the observation of the ^m4^C modification. Nonetheless, peptides corresponding to all of the enzymes predicted to be responsible for methylation in *C*. *jejuni* were detected using proteomic analysis. This study provides the foundation for studies to determine the functions of the putative restriction-modification enzymes in *C*. *jejuni*.

## Supporting Information

S1 Fig Diagram of the *C*. *jejuni* F38011 CJH00185 CDS (CjeF38011ORFAP enzyme). The putative CJH00185 gene is 3491 base pairs (bp) with a frameshift mutation at position 1989 that splits the sequence into two ORFs (light gray and dark gray boxes). The consensus sequences for the modified bases (^m4^C sites) are indicated.
(TIF)

S2 Fig The *C*. *jejuni* F38011 CJH00185 mutant demonstrates characteristics indistinguishable from the wild-type isolate. Panel A) Bacterial growth rates in broth are similar between strains in MH broth with and without 0.05% DOC. Panel B) Bacterial survival over time is similar between strains in MH broth with and without 0.05% DOC, as determined by CFU analysis. Panel C) Invasion of INT 407 cells by *C*. *jejuni* following incubation with INT 407 cells for 30 and 180 min. There was no statistical difference in initial bacterial binding to cells between strains.
(TIF)

S1 Table Unique predicted protein coding sequences (CDSs) between *C*. *jejuni* strains F38011 and NCTC 11168. (XLSX)

S2 Table UniProt proteomic analysis of *C*. *jejuni* F38011 peptides mapped to *C*. *jejuni* NCTC 11168 (CAMJE). (XLSX)

S3 Table Sequest peptides corresponding to *C*. *jejuni* F38011, as determined by LC-MS/MS. (XLSX)

## References

[1] Sebald M, Veron M. Base DNA Content and Classification of Vibrios. Annales de l'Institut Pasteur. 1963;105:897–910. 14098102

[2] Dekeyser P, Gossuin-Detrain M, Butzler JP, Sternon J. Acute enteritis due to related vibrio: first positive stool cultures. The Journal of infectious diseases. 1972;125(4):390–2. 4553081 10.1093/infdis/125.4.390

[3] Black RE, Levine MM, Clements ML, Hughes TP, Blaser MJ. Experimental *Campylobacter jejuni* infection in humans. The Journal of infectious diseases. 1988;157(3):472–9. Epub 1988/03/01 3343522 10.1093/infdis/157.3.472

[4] Blaser MJ, Berkowitz ID, LaForce FM, Cravens J, Reller LB, Wang WL. *Campylobacter* enteritis: clinical and epidemiologic features. Ann Intern Med. 1979;91(2):179–85. Epub 1979/08/01. 380433 10.7326/0003-4819-91-2-179

[5] Crushell E, Harty S, Sharif F, Bourke B. Enteric *Campylobacter*: purging its secrets? Pediatric research. 2004;55(1):3–12. 10.1203/01.PDR.0000099794.06260.71 14605259

[6] Schwerer B. Antibodies against gangliosides: a link between preceding infection and immunopathogenesis of Guillain-Barre syndrome. Microbes Infect. 2002;4(3):373–84. Epub 2002/03/23. 11909748 10.1016/s1286-4579(02)01550-2

[7] Zhang M, Yang X, Liu H, Liu X, Huang Y, He L, et al Genome Sequences of the Guillain-Barre Syndrome Outbreak-Associated *Campylobacter jejuni* Strains ICDCCJ07002 and ICDCCJ07004. Genome announcements. 2013;1(3). 10.1128/genomeA.00256-13 PMC3662821 23704181

[8] Yuki N, Taki T, Inagaki F, Kasama T, Takahashi M, Saito K, et al A bacterium lipopolysaccharide that elicits Guillain-Barre syndrome has a GM1 ganglioside-like structure. The Journal of experimental medicine. 1993;178(5):1771–5. 8228822 10.1084/jem.178.5.1771 PMC2191246

[9] Biggs PJ, Fearnhead P, Hotter G, Mohan V, Collins-Emerson J, Kwan E, et al Whole-genome comparison of two *Campylobacter jejuni* isolates of the same sequence type reveals multiple loci of different ancestral lineage. PloS one. 2011;6(11):e27121 10.1371/journal.pone.0027121 22096527 PMC3214069

[10] Cooper KK, Cooper MA, Zuccolo A, Law B, Joens LA. Complete genome sequence of *Campylobacter jejuni* strain S3. Journal of bacteriology. 2011;193(6):1491–2. 10.1128/JB.01475-10 21217004 PMC3067632

[11] Champion OL, Gaunt MW, Gundogdu O, Elmi A, Witney AA, Hinds J, et al Comparative phylogenomics of the food-borne pathogen *Campylobacter jejuni* reveals genetic markers predictive of infection source. Proceedings of the National Academy of Sciences of the United States of America. 2005;102(44):16043–8. 10.1073/pnas.0503252102 16230626 PMC1276044

[12] Wilson DJ, Gabriel E, Leatherbarrow AJ, Cheesbrough J, Gee S, Bolton E, et al Tracing the source of campylobacteriosis. PLoS genetics. 2008;4(9):e1000203 10.1371/journal.pgen.1000203 18818764 PMC2538567

[13] Kramer JM, Frost JA, Bolton FJ, Wareing DR. *Campylobacter* contamination of raw meat and poultry at retail sale: identification of multiple types and comparison with isolates from human infection. Journal of food protection. 2000;63(12):1654–9. 11131886 10.4315/0362-028x-63.12.1654

[14] Tock MR, Dryden DT. The biology of restriction and anti-restriction. Current opinion in microbiology. 2005;8(4):466–72. 10.1016/j.mib.2005.06.003 15979932

[15] Miller WG, Pearson BM, Wells JM, Parker CT, Kapitonov VV, Mandrell RE. Diversity within the *Campylobacter jejuni* type I restriction-modification loci. Microbiology. 2005;151(Pt 2):337–51. 10.1099/mic.0.27327-0 15699185

[16] Casadesus J, Low D. Epigenetic gene regulation in the bacterial world. Microbiology and molecular biology reviews: MMBR. 2006;70(3):830–56. 10.1128/MMBR.00016-06 16959970 PMC1594586

[17] Raphael RH, Monteville MR, Klena JD, Joens LA, Konkel ME. Interactions of *Campylobacter jejuni* with non-professional phagocytic cells In: JM K, ME K, editors. Campylobacter molecular and cellular biology. Norfolk: Horizon Bioscience; 2005 p. 397–413.

[18] Samuelson DR, Eucker TP, Bell JA, Dybas L, Mansfield LS, Konkel ME. The *Campylobacter jejuni* CiaD effector protein activates MAP kinase signaling pathways and is required for the development of disease. Cell communication and signaling: CCS. 2013;11:79 10.1186/1478-811X-11-79 24144181 PMC3833307

[19] Konkel ME, Monteville MR, Rivera-Amill V, Joens LA. The pathogenesis of *Campylobacter jejuni*-mediated enteritis. Curr Issues Intest Microbiol. 2001;2(2):55–71. 11721281

[20] Stothard P, Wishart DS. Circular genome visualization and exploration using CGView. Bioinformatics. 2005;21(4):537–9. 10.1093/bioinformatics/bti054 15479716

[21] Carver T, Berriman M, Tivey A, Patel C, Bohme U, Barrell BG, et al Artemis and ACT: viewing, annotating and comparing sequences stored in a relational database. Bioinformatics. 2008;24(23):2672–6. 10.1093/bioinformatics/btn529 18845581 PMC2606163

[22] Crooks GE, Hon G, Chandonia JM, Brenner SE. WebLogo: a sequence logo generator. Genome research. 2004;14(6):1188–90. 10.1101/gr.849004 15173120 PMC419797

[23] Weisbrod CR, Hoopmann MR, Senko MW, Bruce JE. Performance evaluation of a dual linear ion trap-Fourier transform ion cyclotron resonance mass spectrometer for proteomics research. Journal of proteomics. 2013;88:109–19. 10.1016/j.jprot.2013.04.009 23590889 PMC3972134

[24] Gardy JL, Laird MR, Chen F, Rey S, Walsh CJ, Ester M, et al PSORTb v.2.0: expanded prediction of bacterial protein subcellular localization and insights gained from comparative proteome analysis. Bioinformatics. 2005;21(5):617–23. 10.1093/bioinformatics/bti057 15501914

[25] Wu CH, Apweiler R, Bairoch A, Natale DA, Barker WC, Boeckmann B, et al The Universal Protein Resource (UniProt): an expanding universe of protein information. Nucleic acids research. 2006;34(Database issue):D187–91. 10.1093/nar/gkj161 16381842 PMC1347523

[26] Konkel ME, Corwin MD, Joens LA, Cieplak W. Factors that influence the interaction of *Campylobacter jejuni* with cultured mammalian cells. Journal of medical microbiology. 1992;37(1):30–7. 1625313 10.1099/00222615-37-1-30

[27] Christensen JE, Pacheco SA, Konkel ME. Identification of a *Campylobacter jejuni*-secreted protein required for maximal invasion of host cells. Mol Microbiol. 2009;73(4):650–62. Epub 2009/07/25. 10.1111/j.1365-2958.2009.06797.x 19627497 PMC2764114

[28] Malik-Kale P, Raphael BH, Parker CT, Joens LA, Klena JD, Quinones B, et al Characterization of genetically matched isolates of *Campylobacter jejuni* reveals that mutations in genes involved in flagellar biosynthesis alter the organism's virulence potential. Applied and environmental microbiology. 2007;73(10):3123–36. 10.1128/AEM.01399-06 17369342 PMC1907099

[29] Konkel ME, Gray SA, Kim BJ, Garvis SG, Yoon J. Identification of the enteropathogens *Campylobacter jejuni* and *Campylobacter coli* based on the *cadF* virulence gene and its product. Journal of clinical microbiology. 1999;37(3):510–7. 9986804 10.1128/jcm.37.3.510-517.1999 PMC84446

[30] Arakawa K, Tomita M. Measures of compositional strand bias related to replication machinery and its applications. Current genomics. 2012;13(1):4–15. 10.2174/138920212799034749 22942671 PMC3269016

[31] Gundogdu O, Bentley SD, Holden MT, Parkhill J, Dorrell N, Wren BW. Re-annotation and re-analysis of the *Campylobacter jejuni* NCTC11168 genome sequence. BMC genomics. 2007;8:162 10.1186/1471-2164-8-162 17565669 PMC1899501

[32] Batchelor RA, Pearson BM, Friis LM, Guerry P, Wells JM. Nucleotide sequences and comparison of two large conjugative plasmids from different *Campylobacter* species. Microbiology. 2004;150(Pt 10):3507–17. 10.1099/mic.0.27112-0 15470128

[33] Pearson BM, Pin C, Wright J, I'Anson K, Humphrey T, Wells JM. Comparative genome analysis of *Campylobacter jejuni* using whole genome DNA microarrays. FEBS letters. 2003;554(1–2):224–30. 14596944 10.1016/s0014-5793(03)01164-5

[34] Dorrell N, Mangan JA, Laing KG, Hinds J, Linton D, Al-Ghusein H, et al Whole genome comparison of *Campylobacter jejuni* human isolates using a low-cost microarray reveals extensive genetic diversity. Genome research. 2001;11(10):1706–15. 10.1101/gr.185801 11591647 PMC311159

[35] Wilson MK, Lane AB, Law BF, Miller WG, Joens LA, Konkel ME, et al Analysis of the pan genome of *Campylobacter jejuni* isolates recovered from poultry by pulsed-field gel electrophoresis, multilocus sequence typing (MLST), and repetitive sequence polymerase chain reaction (rep-PCR) reveals different discriminatory capabilities. Microbial ecology. 2009;58(4):843–55. 10.1007/s00248-009-9571-3 19697077

[36] Duong T, Konkel ME. Comparative studies of *Campylobacter jejuni* genomic diversity reveal the importance of core and dispensable genes in the biology of this enigmatic food-borne pathogen. Current opinion in biotechnology. 2009;20(2):158–65. 10.1016/j.copbio.2009.03.004 19346123 PMC2769087

[37] Clark TA, Murray IA, Morgan RD, Kislyuk AO, Spittle KE, Boitano M, et al Characterization of DNA methyltransferase specificities using single-molecule, real-time DNA sequencing. Nucleic acids research. 2012;40(4):e29 10.1093/nar/gkr1146 22156058 PMC3287169

[38] Feng Z, Fang G, Korlach J, Clark T, Luong K, Zhang X, et al Detecting DNA modifications from SMRT sequencing data by modeling sequence context dependence of polymerase kinetic. PLoS computational biology. 2013;9(3):e1002935 10.1371/journal.pcbi.1002935 23516341 PMC3597545

[39] Krebes J, Morgan RD, Bunk B, Sproer C, Luong K, Parusel R, et al The complex methylome of the human gastric pathogen *Helicobacter pylori* . Nucleic acids research. 2014;42(4):2415–32. 10.1093/nar/gkt1201 24302578 PMC3936762

[40] Choe W, Chandrasegaran S, Ostermeier M. Protein fragment complementation in M.HhaI DNA methyltransferase. Biochemical and biophysical research communications. 2005;334(4):1233–40. 10.1016/j.bbrc.2005.07.017 16040000

[41] Murray IA, Clark TA, Morgan RD, Boitano M, Anton BP, Luong K, et al The methylomes of six bacteria. Nucleic acids research. 2012;40(22):11450–62. 10.1093/nar/gks891 23034806 PMC3526280

[42] Poly F, Threadgill D, Stintzi A. Genomic diversity in *Campylobacter jejuni*: identification of *C*. *jejuni* 81–176-specific genes. Journal of clinical microbiology. 2005;43(5):2330–8. 10.1128/JCM.43.5.2330-2338.2005 15872262 PMC1153751

[43] Gilbert M, Mandrell RE, Parker CT, Li J, Vinogradov E. Structural analysis of the capsular polysaccharide from *Campylobacter jejuni* RM1221. Chembiochem: a European journal of chemical biology. 2007;8(6):625–31. 10.1002/cbic.200600508 17335095

[44] Parkhill J, Wren BW, Mungall K, Ketley JM, Churcher C, Basham D, et al The genome sequence of the food-borne pathogen *Campylobacter jejuni* reveals hypervariable sequences. Nature. 2000;403(6770):665–8. 10.1038/35001088 10688204

[45] Ratel D, Ravanat JL, Charles MP, Platet N, Breuillaud L, Lunardi J, et al Undetectable levels of N6-methyl adenine in mouse DNA: Cloning and analysis of PRED28, a gene coding for a putative mammalian DNA adenine methyltransferase. FEBS letters. 2006;580(13):3179–84. 10.1016/j.febslet.2006.04.074 16684535

[46] Malik-Kale P, Parker CT, Konkel ME. Culture of *Campylobacter jejuni* with sodium deoxycholate induces virulence gene expression. Journal of bacteriology. 2008;190(7):2286–97. Epub 2008/01/29. 10.1128/JB.01736-07 18223090 PMC2293209

