# Supplementary figures and images for: Analysis of the Campylobacter jejuni Genome by SMRT DNA Sequencing Identifies Restriction-Modification Motifs
Source: PLoS One. 2015 Feb 19;10(2):e0118533. doi: 10.1371/journal.pone.0118533 (PMC4335053; doi:10.1371/journal.pone.0118533)

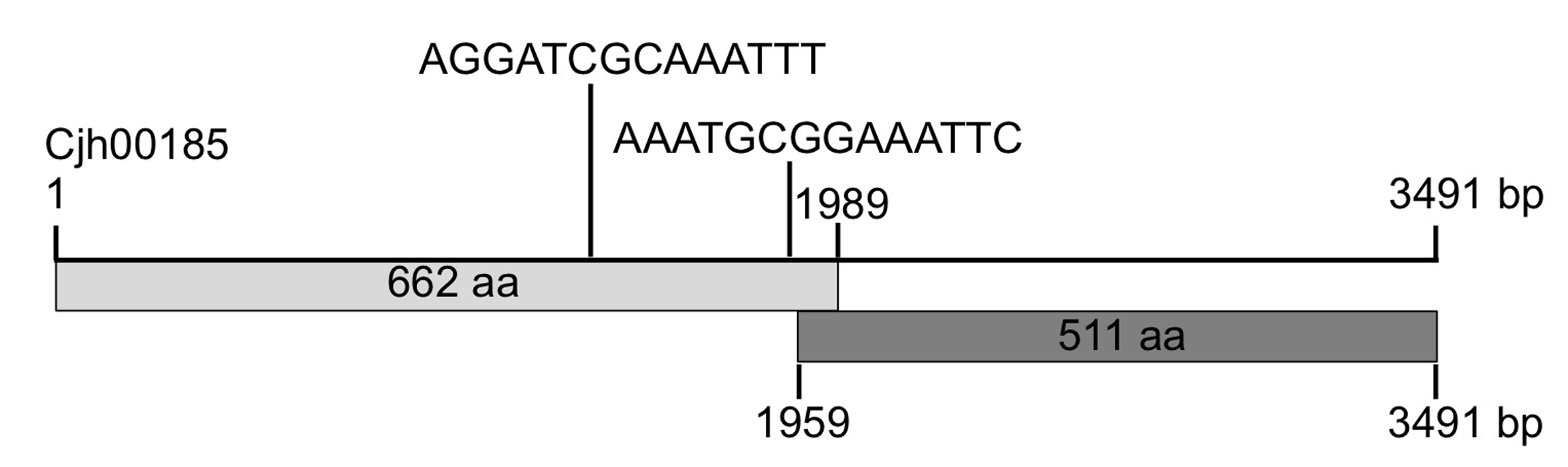

Supplement: S1 Fig — The putative CJH00185 gene is 3491 base pairs (bp) with a frameshift mutation at position 1989 that splits the sequence into two ORFs (light gray and dark gray boxes). The consensus sequences for the modified bases (m4C sites) are indicated. (TIF) [file pone.0118533.s001.tif]

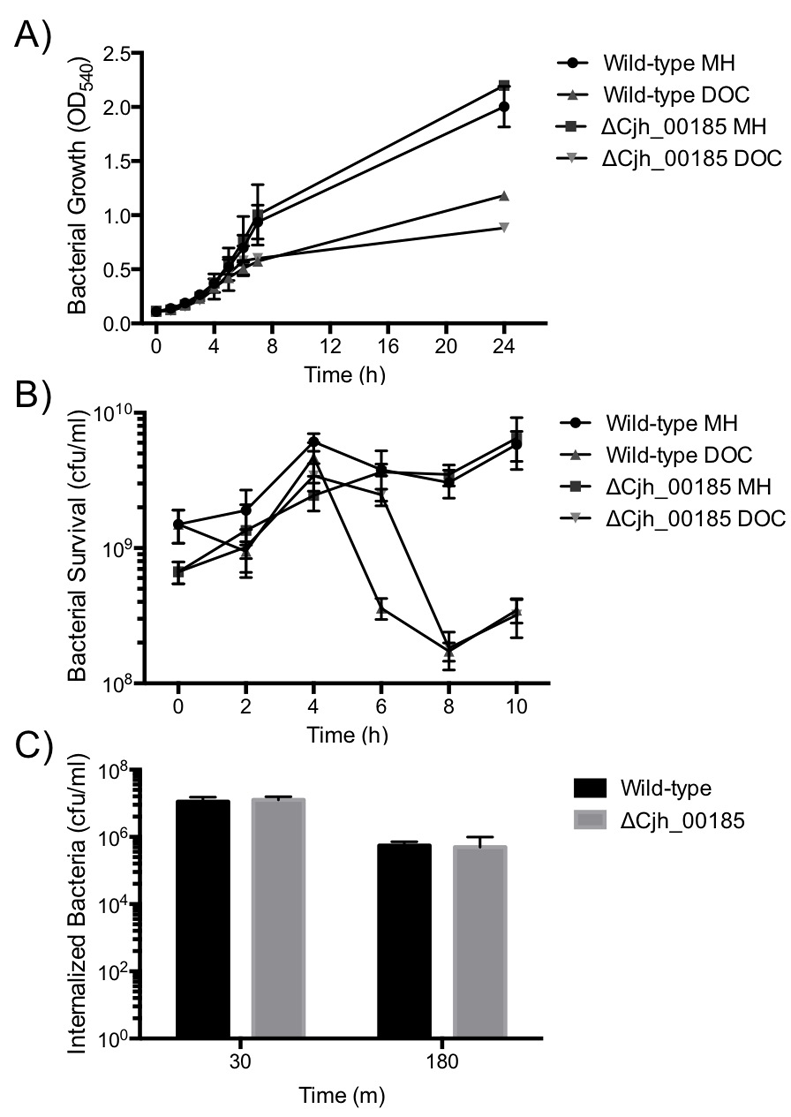

Supplement: S2 Fig — Panel A) Bacterial growth rates in broth are similar between strains in MH broth with and without 0.05% DOC. Panel B) Bacterial survival over time is similar between strains in MH broth with and without 0.05% DOC, as determined by CFU analysis. Panel C) Invasion of INT 407 cells by C. jejuni following incubation with INT 407 cells for 30 and 180 min. There was no statistical difference in initial bacterial binding to cells between strains. (TIF) [file pone.0118533.s002.tif]
